# Complete Genome Sequencing of *Erwinia phyllosphaerae* ZX-13, a Novel Biocontrol Agent to Against the Stem Blight Pathogen *Pseudocryphonectria elaeocarpicola* in *Elaeocarpus* spp.

**DOI:** 10.3390/microorganisms13122678

**Published:** 2025-11-25

**Authors:** Huayi Huang, Yi Zhao, Lili Hu, Chenglong Gao, Shiying Chen, Danyang Zhao

**Affiliations:** Guangdong Provincial Key Laboratory of Silviculture, Protection and Utilization, Guangdong Academy of Forestry, Guangzhou 510520, China; huanghy@sinogaf.cn (H.H.);

**Keywords:** *Erwinia phyllosphaerae*, genome sequencing, antagonistic ability, the stem blight of *Elaeocarpus* spp., *Pseudocryphonectria elaeocarpicola*

## Abstract

The stem blight disease on *Elaeocarpus* spp. was newly identified in 2022 on *E. rugosus* and *E. hain* in Guangzhou, representing a serious stem disease that causes branch rot and cankers in *Elaeocarpus* species and can lead to whole-plant death in severe cases. Strain ZX-13 was isolated from *Elaeocarpus rugosus* stems and showed excellent antagonism against the *Elaeocarpus* stem blight pathogen *Pseudocryphonectria elaeocarpicola.* Based on morphological characteristics, physiological and biochemical results, and molecular biology analyses, the strain was identified as *Erwinia phyllosphaerae.* Whole-genome sequencing and annotation indicate that strain ZX-13 has a high-quality draft assembly of 4,686,433 bp, with a GC content of 53.85%, encoding 4189 genes, 86 tRNA, 22 rRNA, and 110 ncRNA. Eight antifungal enzymes from the GH family may be crucial factors to its antagonistic activity. Using antiSMASH 7.1.0, the ZX-13 genome predicted eight BGCs (RiPPs, NRPs, terpenes, etc.), with one showing 100% similarity to carotenoid biosynthesis. Novel candidates include an O-antigen, hassallidin C, and lankacidin C. The study identified *Erwinia phyllosphaerae* ZX-13 as an antagonistic bacterial strain for the first time, indicating substantial potential for biocontrol applications.

## 1. Introduction

*Elaeocarpus*, belonging to the Elaeocarpaceae family, is a large genus with approximately 550 species of evergreen trees. It is widely distributed across tropical and subtropical regions. *Elaeocarpus* plants grow rapidly, with straight trunks. In addition to their ornamental value for landscaping and urban green space, *Elaeocarpus* species are also highly valued for their economic and medicinal benefits. For example, the wood of most *Elaeocarpus* species can be made into furniture, their fruits are edible, and their seeds are used as materials for prayer beads, bracelets, and necklaces [1,2]. Studies have shown that extracts from *Elaeocarpus* plants are rich in various polyphenolic compounds, which exhibit antioxidant activity and can inhibit enzymes related to human physiological health [1,3,4]. The stem blight of *Elaeocarpus* is a serious branch disease caused by the fungus *Pseudocryphonectria elaeocarpicola*. It severely damages *E. rugosus, E. hainanensis,* and *E. sylvestris*, leading to branch decay, dieback, and plant death [5,6].

Currently, no cost-effective methods for controlling and preventing the stem blight of *Elaeocarpus* have been found. Using biocontrol bacteria to inhibit it may help address this dilemma. Biological control bacteria are widely used in agriculture and forestry to prevent various plant diseases and improve crop yields and have advantages such as environmental friendliness, low toxicity, and sustainability. The biological control bacteria successfully applied in plant disease prevention mainly originate from the *Bacillus* spp. and *Pseudomonas* spp. [7,8,9,10,11,12]. Additionally, *Serratia*, *Pasteuria* and *Agrobacterium* are also commonly used as biocontrol agents [13,14,15,16]. For example, *B. subtilis* strain MBI 600 demonstrates inhibitory effects against a variety of plant pathogens and growth promotion on cucumber [9]. *B. megaterium* 6A and *B. subtilis* 11A can significantly inhibit the germination of *Puccinia striiformis* f. sp. *tritici* spores. Both indoor experiments and field trials with *B. megaterium* 6A effectively controlled wheat stripe rust [17].

Biocontrol agents offer sustainable alternatives to chemical pesticides, yet the potential of *Erwinia* spp. as antagonists remains underexplored. Although *Erwinia* spp. are widely recognized as pathogenic and epiphytic bacteria (e.g., *E. papayae*, *E. piriflorinigrans*, *E. uzenensis*, *E. gerundensis*) [18,19,20,21], their limited use as biocontrol agents presents a unique opportunity: they may reduce the selective pressure for resistance in target pathogens compared with conventional antagonists. An increasing number of researchers are utilizing whole-genome sequencing technology to explore the genomic information of antagonistic strains and predict their functions [7,22,23]. Accordingly, *E. phyllosphaerae* ZX-13, isolated from *Elaeocarpus* stems and exhibiting strong antibacterial activity, was selected as the research object; its genome was sequenced and analyzed in detail against public databases (NR, Swiss-Prot, COG, GO, KEGG, CAZy), and potential bacteriocins were predicted using antiSMASH, providing a bioinformatics foundation to guide the development of ZX-13 as a biocontrol candidate.

## 2. Materials and Methods

### 2.1. Evaluation of the Antibacterial Activity of Experimental Strain

The experimental strain ZX-13 was isolated from the stem of samples of *Elaeocarpus rugosus* in Guangzhou, Guangdong Province, China. The strain was deposited in the Guangdong Microbial Culture Collection Center (GDMCC No: 66800, https://www.gdmcc.net/, accessed on 21 August 2025) and Guangdong Academy of Forestry (GDAF, http://www.sinogaf.cn/, accessed on 21 July 2022). The bacteria were cultured in Luria-Bertani agar (LB) medium (LB: 10 g/L tryptone, 5 g/L yeast extract, 10 g/L NaCl, and 17 g/L agar) for 1 day at 28 °C in the dark. The evaluation of antagonistic bacteria was performed using the dual culture method [24]. A newly grown colony of *P. elaeocarpicola* (6 mm) was inoculated at the center of a PDA plate and cultured at 28 °C in an incubator for 24 h. Bacteria were then streaked inoculated at a distance of 2 cm from the mycelium, and the plates were incubated at 28 °C for 5 days. The width of the inhibition zone was measured.

### 2.2. Morphological and Physiological-Biochemical Analysis

Strain ZX-13 was preliminarily identified by using morphological and physiological-biochemical analysis. The preliminary identification was performed by observing colony morphology features such as shape, color, edge, and moistness/dryness. The ultra-structural details of the bacterial cells were examined using a SU8100 scanning electron microscope (SEM; Hitachi, Tokyo, Japan), and cell size measurements were conducted. A reference was used for physiological and biochemical identification of the strain [25].

### 2.3. The Biocontrol Effect of Strain ZX-13 Fermentation Broth on Elaeocarpus Species Stem Blight Under Laboratory Conditions

To evaluate the biocontrol efficacy of strain ZX-13 against *Elaeocarpus* spp. stem blight caused by *P. elaeocarpicola*, pathogenicity tests were conducted on 20 healthy two-year-old uniformly grown *E. hainanensis* seedlings. The inoculation methods and related data calculation refer to the research methods of Dai et al. [26]. The surface of the main stem, 10 to 30 cm above the base of the *E. hainanensis*, was rinsed with clean water, then the surface was sterilized with 75% alcohol and rinsed three times with sterile water. The main stem was scalded with a soldering iron in a circular area 6 mm in diameter, located 20 cm above the twig base. In the experimental group, 2 mL of strain ZX-13 bacterial suspension (1.0 × 10^5^ cfu/mL) was sprayed onto the scalded and surrounding area, followed by inoculation with *P. elaeocarpicola* mycelium, which was then wrapped with wetted sterile cotton and covered with preservative film for humidity preservation (ZX + PE). The negative control group consisted of two subgroups: one was first sprayed with sterile water and then inoculated with sterile PDA (WA + PD), and the other was first sprayed with sterile water and then inoculated with *P. elaeocarpicola* mycelium (WA + PE). The positive control involved spraying 5 μg/mL of 96.8% difenoconazole along with *P. elaeocarpicola* mycelium (DF + PE). Five plants were inoculated in each treatment group. After 2 days post-inoculation, the degreasing cotton and preservative film were removed. After 4 days post-inoculation, the twigs were observed for disease development. To measure the lesion areas, the length and width of lesions was measured, and the lesion area was calculated according to the elliptical area formula:

Lesion Area (S) = π × Length × Width/4

The control effect was computed by the following equation:

Control Effect (%) = 100 × [(S_WA+PE_ − S _WA+PD_) − (S_ZX+PE_ − S_WA+PD_)]/(S_WA+PE_ − S_WA+PD_)

where S_WA+PE_, S_WA+PD_, and S_ZX+PE_ represent the lesion areas of WA + PE (mm^2^), WA + PD (mm^2^), and ZX + PE (mm^2^), respectively.

### 2.4. Genome Sequencing, Assembly and Annotation

A genomic DNA sample was isolated from the cell pellets with a Bacteria DNA Kit (OMEGA, Norcross, GA, USA) according to the manufacturer’s instructions, and quality control was subsequently carried out on the purified DNA samples. Sequencing was performed by Qingke Biotech (Beijing, China). We employed a hybrid sequencing strategy combining Illumina short reads and PacBio long reads. First, quality-trimmed Illumina reads were assembled using ABySS 2.2.0 with multiple k-mers to generate a primary assembly (http://www.bcgsc.ca/platform/bioinfo/software/abyss). Then, this Illumina-based assembly was used to correct the PacBio long reads, which were subsequently assembled using Canu (https://github.com/marbl/canu). The final assembly was improved by closing local gaps with GapCloser (https://sourceforge.net/projects/soapdenovo2/files/GapCloser/), and the genome structure was visualized with Circos v0.64 (http://circos.ca/).

The gene model of strain ZX-13 was predicted using GeneMark. The predicted protein sequences were then functionally annotated by performing BLASTP searches against several databases, including the non-redundant (NR) protein database at NCBI, KEGG (http://www.genome.jp/kegg/), GO (http://www.geneontology.org/), COG (http://www.ncbi.nlm.nih.gov/COG), Swiss-Prot (https://www.uniprot.org/downloads#uniprotkblin) and CAZy (http://www.cazy.org/). In addition, tRNAs were identified using the tRNAscan-SE (v1.23, http://lowelab.ucsc.edu/tRNAscan-SE), and rRNAs were determined using the RNAmmer (v1.2, http://www.cbs.dtu.dk/services/RNAmmer/). 

All software versions and corresponding resources cited were accessed on 20 September 2022.

### 2.5. Phylogenetic Analyses

The phylogenetic trees based on entire genome sequences were constructed with some species of the genus *Erwinia* by the approximate maximum-likelihood method using OrthoFinder v2.5.4 [27]. Based on the single-copy ortholog approach, phylogenetic analysis can analyze more genes, including those that may be lost or diverge in some species, which helps to understand gene functions and evolutionary relationships. The protein files of 11 strains were placed in one folder and run in OrthoFinder v2.5.4 to identify orthologs and build the species tree. OrthoFinder handles clustering, alignment, single-copy ortholog extraction, concatenation, and tree inference. It uses MAFFT for aligning single-copy orthologs and IQ-TREE for the maximum-likelihood tree with bootstrap. The final output is the phylogenetic tree file. The 16S rDNA sequence of the strain was first analyzed by BLAST in the NCBI database (https://blast.ncbi.nlm.nih.gov/Blast.cgi, accessed on 4 March 2025). Molecular evolutionary analysis and phylogenetic tree construction of the 16S rDNA sequence data were performed using MEGA 10.0 software. Whole-genome sequences of the strains were downloaded from the GenBank database (https://www.ncbi.nlm.nih.gov/genome, accessed on 4 March 2025), and genome sequences are listed in Table 1. The sequences used for 16S rDNA phylogenetic tree construction are extracted from the whole genome.

### 2.6. ANI and GGDC Analyses

The average nucleotide identity (ANI) and genome-to-genome distance calculation (GGDC) analyses employed two independent methods of digital DNA–DNA hybridization (dDDH), namely ANI and GGDC, to estimate the overall similarity between the genomes of the two strains. ANI analysis was performed using the online platform JspeciesWS (http://jspecies.ribohost.com/jspeciesws/, accessed on 17 March 2025). The GGDC 3.0 program provided by the German Collection of Microorganisms and Cell Cultures (DSMZ; Braunschweig, Germany) was used for GGDC analysis (DSMZ; Braunschweig, Germany; http://ggdc.dsmz.de, accessed on 17 March 2025). The heatmaps of the ANI and dDDH values were generated using TBtools v2.056. 3 [28].

### 2.7. Discovery of Bioactive Compound

Prediction and analysis of bioactive compounds were performed using the online database antiSMASH (http://antismash.secondarymetabolites.org/, accessed on 10 March 2025) to predict the biosynthetic gene clusters of secondary metabolites in strain ZX-13.

### 2.8. Photographs Editing and Statistical Analysis

Photographs were edited using Adobe Photoshop CS6, and data analyzed and visualized using WPS Excel 2025.

## 3. Results

### 3.1. The Biocontrol Efficacy of Strain ZX-13

**Figure 1:**
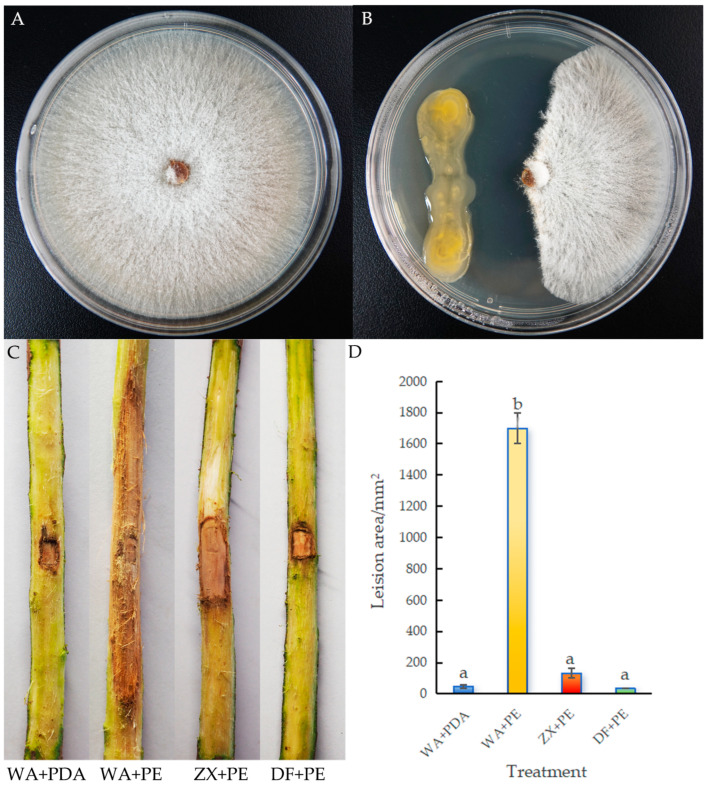
Antagonistic activity of ZX-13 against *P. elaeocarpicola*. (**A**) The colony of *P. elaeocarpicola* grown on a PDA plate for 5 days. (**B**) The inhibition zone after co-culturing strain ZX-13 and *P. elaeocarpicola* on a PDA plate for 5 days. (**C**) Pathogenicity of *P. elaeocarpicola* under different treatments. (**D**). Lesion area of *P. elaeocarpicola* under different treatments. Different letters represent significant differences (*p* < 0.05), while the same letters indicate no significant differences.

Through a dual culture assay, strain ZX-13 showed an excellent antagonistic effect against the pathogen. The colony diameter of *P. elaeocarpicola* grown on a PDA plate for 5 days was 84.00 mm (Figure 1A). After co-culturing strain ZX-13 and *P. elaeocarpicola* on a PDA plate for 5 days, the inhibition zone width was 10.17 mm (Figure 1B). After 4 days of inoculation, the *E. hainanensis* seedling stems sprayed with sterile water and sterile PDA (WA + PDA) remained green and healthy. In contrast, seedlings inoculated with water and *P. elaeocarpicola* (WA + PE) exhibited typical disease symptoms, with stem blight and browning beginning to appear. Seedlings inoculated with fermentation broth of strain ZX-13 and *P. elaeocarpicola* (ZX + PE) showed a much smaller lesion area than WA + PE. Additionally, seedlings inoculated with 96.8% difenoconazole and *P. elaeocarpicola* (DF + PE) showed no symptoms, similar to the WA + PDA treatment group (Figure 1C). The WA + PE treatment produced a significantly different lesion area compared with the other three treatments, while the difference in lesion area between ZX + PE treatment and WA + PDA and DF + PE treatments was not significant (*p* < 0.05). This indicates that strain ZX-13 has a significant antifungal effect (Figure 1D). These results indicate that the strain ZX-13 exhibits antagonistic activity against *P. elaeocarpicola* under laboratory conditions, with a control efficacy of 94.85%.

### 3.2. Morphological Characteristics and Physiological and Biochemical Properties of Strain ZX-13

**Figure 2:**
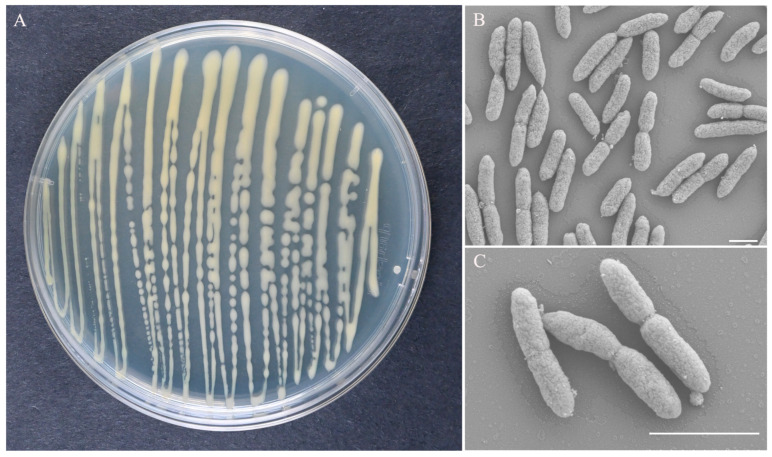
Morphological observations of strain ZX-13. (**A**) Colony morphology on 90 mm LB agar plate, (**B**,**C**) Rod-shaped bacteria were visualized using scanning electron microscopy. Scale bars: 0.002 mm.

The colonies of strain ZX-13 grown on LB agar medium were off-white to gray-yellow, oblong, smooth, moist and slightly convex, and had an entire margin. Colony size was 0.3–2.2 (av. = 1.35) mm (Figure 2A). The cells were rod-shaped, 0.50–0.55 (av. = 0.53) µm wide, and 2.28–2.77 (av. = 2.52) µm long (Figure 2B,C).

Compared with the model strain *E. phyllosphaerae* CMYE1T, most physiological and biochemical characteristics are shared [27]. Strain ZX-13 is negative for arginine dihydrolase, citrate assimilation, and indole production, while strain CMYE1T is positive. Strain ZX-13 is positive for D-glucose, whereas strain CMYE1T is negative (Table 2). Strain ZX-13 has a significantly higher C16:0 content than strain CMYE1T; the relative amounts of C12:0, C14:0, and C17:0 differ only slightly between the two strains, indicating little difference in short-chain saturated fatty acids. For cyclic fatty acids, strain ZX-13 and CMYE1T show notable differences in C17:0 cyclo and C19:0 cyclo ω8c, with C17:0 cyclo at 7.84 vs. 13.5 and C19:0 cyclo ω8c at 0.67 vs. 1.8, suggesting greater accumulation of cyclic fatty acids in strain CMYE1T, which may affect membrane fluidity and permeability. Values for summed features 2/3/8 also differ between the two strains, potentially reflecting differences in cell membrane lipid composition and metabolic state (Table 3).

### 3.3. Genome Feature Analysis and Function Annotation

**Figure 3:**
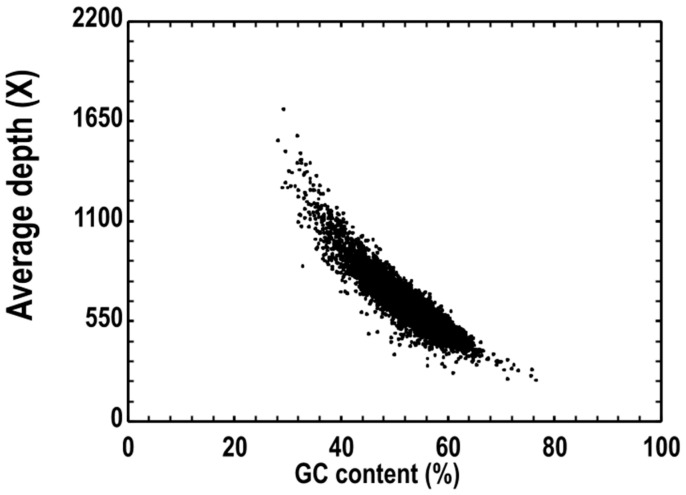
Correlation between GC content of samples and sequencing depth.

The assembled genome for the bacterial sample comprises a single contig with a total length of 4,686,433 bp. The N50 length equals the total assembly length (4,686,433 bp), and the assembly exhibits a read coverage of approximately 1767.19× with a GC content of 53.85%. The fraction of undetermined nucleotides (N rate) is 0% (Table 4). Coverage depth is stable in high-depth regions: across low- to mid-GC content (roughly 20–60%), most regions have coverage concentrated in a higher range (200–1000+), suggesting that sequencing data provide sufficient support for the majority of the genome, facilitating accurate gene annotation and assembly validation (Figure 3).

N50 is defined as the length L for which the collection of all scaffolds of length ≥ L contains at least 50% of the total assembly length. N rate is the proportion of ambiguous bases (N) in the assembly.

**Figure 4:**
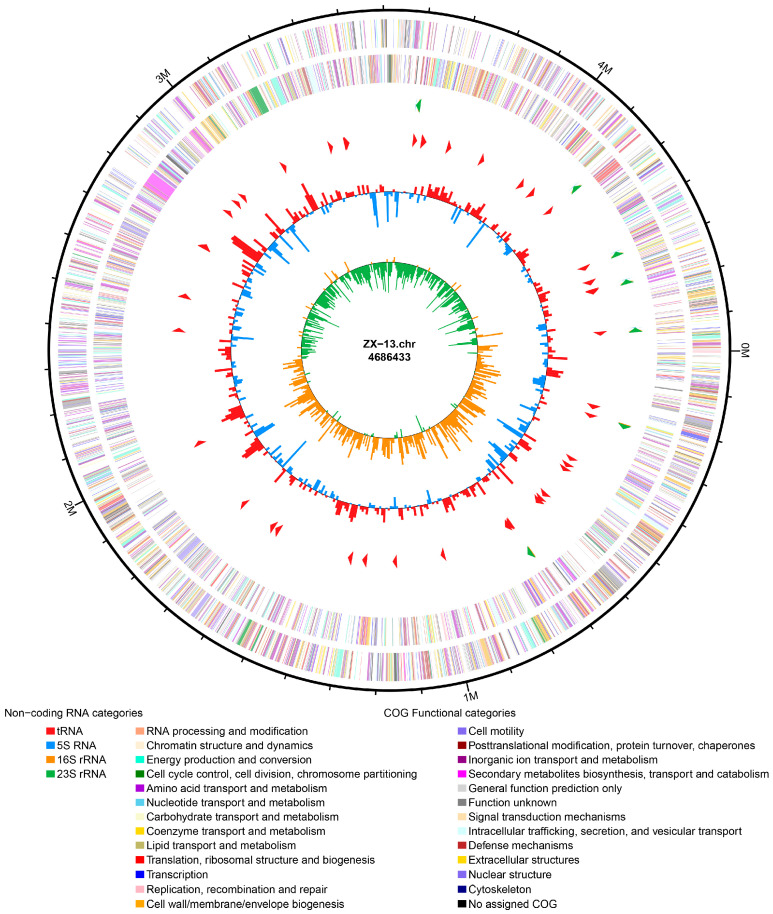
Circular representation of the genome of ZX-13. From the outside to the inside, the legends are as follows: CDS on the positive and negative strands, and the different colors indicate the functional classification of the different COGs of CDS; rRNAs and tRNAs; GC content; GC skew.

**Figure 5:**
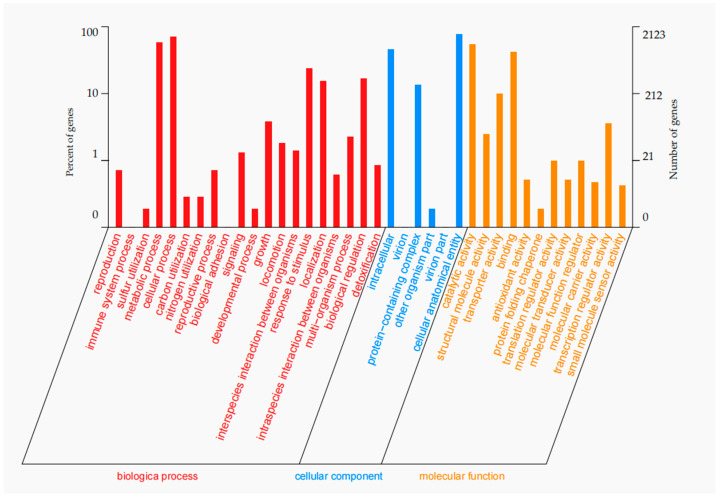
The terms annotated using the Gene Ontology database.

**Figure 6:**
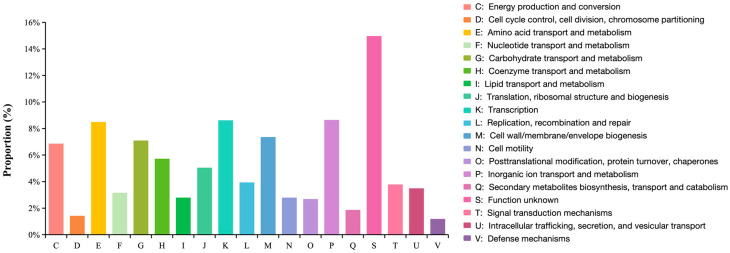
COG categories of strain ZX-13.

The genome data have been deposited in Genbank under the accession number of JBRILN000000000. ZX-13 achieves a high-quality draft genome of 4,686,433 bp (GC content: 53.85%), annotated to include 86 tRNAs, 22 rRNAs, and 110 ncRNAs, indicating robust gene content recovery. The total number of protein-coding genes is 4187. In the NR, GO, KEGG, COG, and SwissProt databases, the numbers are 4028, 2123, 2708, 3540, and 3214, respectively (Figure 4; Table 5). According to GO annotations, based on being divided into 38 functional groups, the genes involved in biological processes are the most abundant, and the number of genes in the cellular anatomical entity is the largest, reaching 1628 (Figure 5). The protein-coding genes of ZX-13 were classified into 19 different functional protein ortholog groups. Inorganic ion transport and metabolism (9%), transcription (9%), and amino acid transport and metabolism (8%) are the top three enriched categories (Figure 6). However, approximately 15% of the protein-coding genes have yet to be characterized in detail.

**Figure 7:**
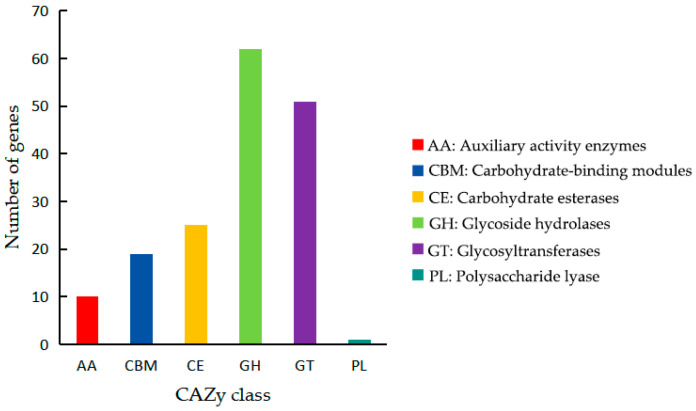
Functional feature genes annotated based on CAZy database.

Analysis using the CAZy database identified 168 carbohydrate-active enzymes (CAZymes), including 10 auxiliary activity enzymes (AA), 19 carbohydrate-binding modules (CBM), 25 carbohydrate esterases (CE), 62 glycoside hydrolases (GH), 51 glycosyltransferases (GT), and 1 polysaccharide lyase (PL) (Figure 7). Among these enzymes, the identification of specific glycoside hydrolases known to target fungal cell walls—such as those with predicted functions as chitinases (GH18, GH19, GH23), and others (GH1, GH3, GH5, GH13, GH32)—suggests a genetic potential for antifungal activity [7,8]. This genomic analysis provides a foundation for the hypothesis that strain ZX-13 may possess antifungal mechanisms, revealing the diversity of its CAZymes and offering guiding the subsequent development of antifungal and biocontrol agents.

### 3.4. Identification of Strain ZX-13 as Erwinia phyllosphaerae Using 16S rRNA Gene and Whole-Genome Phylogeny

**Figure 8:**
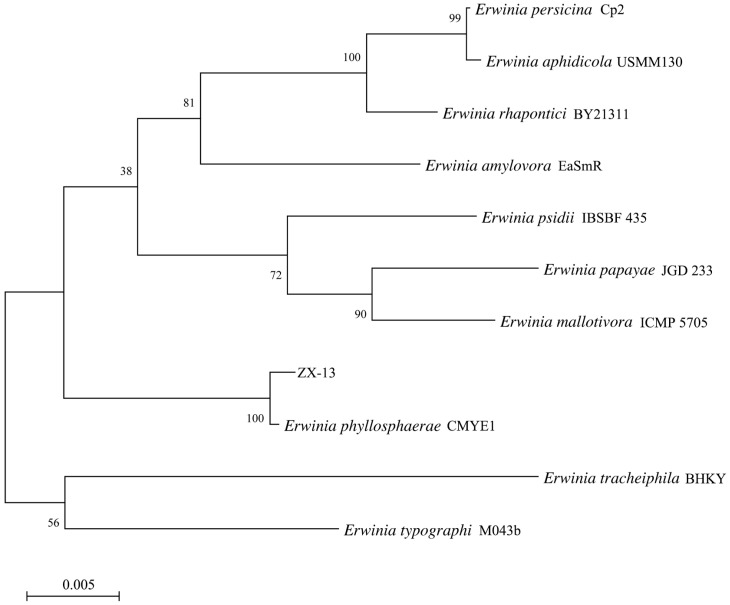
Phylogenomic Neighbor Joining tree (Kimura 2-parameter model, 500 bootstraps) of ZX-13 based on 16 S rDNA sequences. The numbers in each branch point denote the percentages supported by bootstrap. Bar = 0.5% nucleotide divergence. Strain accession numbers are provided in Table 1.

**Figure 9:**
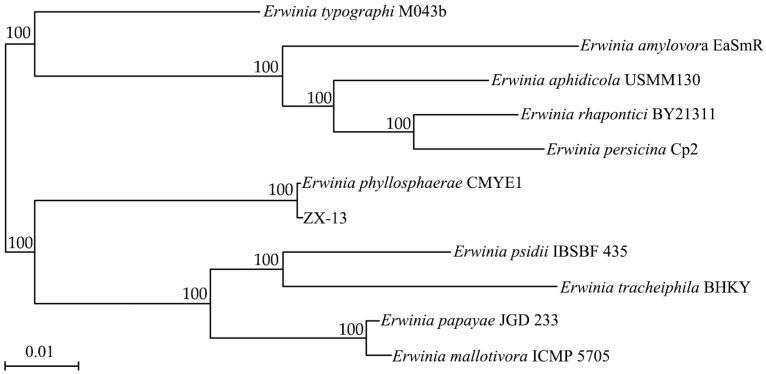
Maximum-likelihood phylogenetic tree of ZX-13 based on whole-genome sequences. The numbers in each branch point denote the percentages supported by bootstrap. Bar = 1% nucleotide divergence. Strain accession numbers are provided in Table 1.

From a molecular biology perspective, to identify strain ZX-13, the 16S rRNA gene analysis was conducted first. BLAST results show that the strain closely matches *E*. *phyllosphaerae* CMYE1, achieving 100% coverage and 99.8% identity. To further elucidate the evolutionary relationship between ZX-13 and the major species of the genus *Erwinia*, a phylogenetic analysis based on 16S rDNA was performed. As shown in Figure 8, ZX-13 and *E*. *phyllosphaerae* CMYE1T clustered into a single branch with a bootstrap value of 100% based on the Neighbor Joining method. Furthermore, a phylogenetic analysis based on the whole-genome sequence also indicated that ZX-13 is most closely related to *E. phyllosphaerae* CMYE1T, with a bootstrap value of 100% (Figure 9).

### 3.5. ANI and GGDC in Genomic Distance Estimation

**Figure 10:**
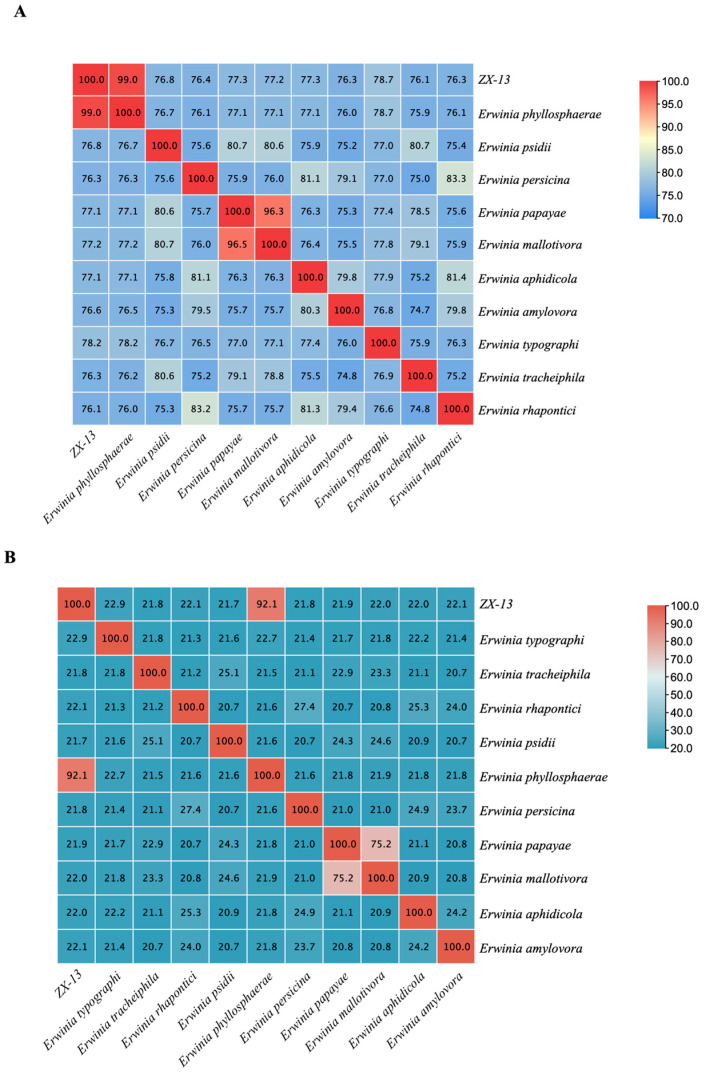
Heatmap analyses of ANI (**A**) and dDDH (**B**) values (%). The color represents the ANI or dDDH values. The red indicates the highest ANI or DDH value, and the blue indicates the least ANI or dDDH value.

The heatmap analysis of ANI values is shown in Figure 10A. The ANI value between ZX-13 and the *E. phyllosphaerae* reference genome is 98.96%, above the species delineation threshold of 95%. The ANI values between ZX-13 and other strains are below 91%. The heatmap analysis based on the predicted dDDH values is shown in Figure 10B. For ZX-13 and *E. phyllosphaerae*, the predicted dDDH value is 92.1%, well above the proposed species cutoff of 70%. The dDDH values between ZX-13 and other strains are below 42%. Based on the results of the comprehensive ANI value and dDDH value, it can be considered that ZX-13 belongs to the same species as *E. phyllosphaerae*.

### 3.6. Bioactive Compound Prediction

Using the antiSMASH 7.1.0 online database, eight biosynthetic gene clusters (BGCs) were predicted in the genome of ZX-13, including ribosomally synthesized and post-translationally modified peptides (RiPPs), nonribosomal peptides (NRPs), terpenes, and others (Table 6). Among them, a single putative gene cluster exhibited 100% similarity to the carotenoid biosynthesis pathway. The corresponding novel secondary metabolite candidates also include an O-antigen, hassallidin C, and lankacidin C.

## 4. Discussion

The stem blight of *Elaeocarpus* spp. caused by *P. elaeocarpicola* has been observed in *E. rugosus, E. hainanensis*, and *E. sylvestis*. In Guangdong Province’s Pearl River Delta region, the occurrence and impact of *Elaeocarpus* spp. disease are particularly severe. According to investigations conducted by our laboratory in 2022, the infection rate in some areas of Guangzhou reached as high as 88.89% to 100%, with a mortality rate of 15.38% to 62.5%. This has caused substantial landscape, ecological, and economic losses [5,6]. In this study, a strain named ZX-13 was isolated from *E. rugosus* stems. Antagonistic test and pathogenicity test results indicated that the strain ZX-13 could significantly inhibit the growth of the pathogenic fungus. Based on morphological characteristics, physiological and biochemical tests, and molecular biological analyses, it was identified as *E. phyllosphaerae*.

There has been limited research on *Erwinia* genus for biological control. Currently, *E. herbicola* (syn. *Pantoea vagans)* is used for the control of fire blight in apple and pear, wheat *Fusarium* wilt, and blue mold in mandarin [29,30,31]. Additionally, strain EUS78 of *E. persicina* can inhibit the growth of *Salmonella enterica* [32]. In this study, *E. phyllosphaerae* is first reported as a biological control bacterium. Its model strain is *E. phyllosphaerae* CMYE1, which was isolated from the phyllosphere of pomelo (*Citrus maxima*) in Guangdong Province. The genome sequence length of strain ZX-13 is approximately 4.47 Mb, slightly shorter than that of strain CMYE1T, which is 4.73 Mb. Both strains have the same GC content at about 53.8%. Distinct phenotypic differences were observed between strain ZX-13 (cultured on LB medium) and the type strain CMYE1T (reference data from R2A medium), particularly in fatty acid composition and biochemical traits such as arginine dihydrolase activity, citrate assimilation, indole production, and D-glucose fermentation. While these variations likely stem from genuine divergence in metabolic pathway, genomic architecture, and transcriptional regulation, it is important to note that the differing cultivation conditions may have also contributed. Therefore, the phenotypic comparisons should be interpreted with caution, and primary taxonomic conclusions are more robustly drawn from genomic data.

The eight predicted biosynthetic gene clusters (BGCs) of metabolites produced by *E. phyllosphaerae* ZX-13 include secondary metabolites such as carotenoids, O-antigen, hassallidin C, lankacidin C, and others [Table 6]. Carotenoids, through mechanisms such as antioxidant activity, antimicrobial effects, and regulation of plant immunity, contribute to enhancing plant resistance to diseases [33]. Genome sequencing of the biocontrol bacterium *E. herbicola* C9-1 has also revealed this antimicrobial compound [34]. O-antigen is a polysaccharide component of bacterial outer membrane lipopolysaccharide (LPS) that can protect bacteria from host antibodies, antimicrobial peptides, and other immune responses [35]. Hassallidin C is a class of natural products with antibiotic activity, belonging to polysaccharide-ester derivatives [36]. Lankacidin C is a key factor in biosynthesis produced via polyketide synthase (PKS) and ribosomal peptide synthase (NRPS) pathways, and it possesses broad-spectrum antibacterial activity [37]. It is worth noting that, aside from carotenoids which showed a 100% similarity in the antiSMASH database comparison, the similarity of other gene clusters was not high. Further validation remains to be carried out in follow-up experiments, such as LC-MS analysis and gene expression studies. This suggests that the strain *E. phyllosphaerae* ZX-13 may have the potential to produce more novel antibacterial metabolites, offering significant prospects for applications in agriculture and forestry.

In this study, the whole-genome sequencing and annotation of *E. phyllosphaerae* ZX-13 revealed key genes and pathways related to growth and metabolism. Future research could integrate metabolomics to elucidate the biosynthetic pathways of antifungal compounds and optimize metabolic flux to increase the production of antifungal products. Additionally, the organism’s allergenicity, survival, and dissemination in different environments (soil, water bodies, and airborne particulates) and potential impact on human and animal health should be assessed.

## 5. Conclusions

The study isolated *E. phyllosphaerae* ZX-13 from *E. rugosus* stems. Both antagonistic and pathogenic activity assays indicated that ZX-13 inhibits the growth of the pathogen *P. elaeocarpicola*, which causes *Elaeocarpus* spp. stem blight disease. Whole-genome sequencing and functional annotation indicated that ZX-13 carries eight enzymes with potential antifungal activity in the GH family and eight gene clusters related to the biosynthesis of antimicrobial metabolites, which may be key to its antagonistic effects. These findings provide a bioinformatic foundation for the development and application of novel antimicrobial agents and offer theoretical support for further study of the antimicrobial properties of *E. phyllosphaerae* ZX-13.

## Figures and Tables

**Table 1:** The GenBank accession numbers of genome sequences for *Erwinia* spp.

Strain	GenBank Accession Numbers
*Erwinia amylovora* EaSmR	GCA_043228865.1
*Erwinia aphidicola* USMM130	GCA_037149315.1
*Erwinia mallotivora* ICMP 5705	GCA_042432085.1
*Erwinia papayae* JGD 233	GCA_040741485.1
*Erwinia persicina* Cp2	GCA_019844095.1
*Erwinia phyllosphaerae* CMYE1	GCA_019132875.1
*Erwinia psidii* IBSBF 435	GCA_003846135.1
*Erwinia rhapontici* BY21311	GCA_020683125.1
*Erwinia tracheiphila* BHKY	GCA_021365465.1
*Erwinia typographi* M043b	GCA_000773975.1

**Table 2:** Physiological and biochemical characteristics of strains ZX-13 and CMYE1T.

Characteristic	ZX-13	CMYE1T
Growth at 4 °C	+	+
Hydrolysis of skimmed milk	+	+
Esterase lipase	−	−
Catalase test	+	/
Oxidase test	−	/
Production of hydrogen sulfide	−	/
Voges–Proskauer reaction	+	/
Phenylalanine deaminase test	−	/
β-Galactosidase	+	+
β-Glucosidase	+	+
Arginine dihydrolase	−	+
Assimilation of citrate	−	+
Indole production	−	+
Fermentation of:		
D-xylose	+	+
Inositol	+	+
Arbutin	+	+
Salicin	+	+
Maltose	−	−
Xylitol	−	−
Gentiobiose	+	+
Lyxose	−	−
D-Arabitol	+	+
Gluconate	−	−
2-Keto-gluconate	−	−
D-Glucose	+	−
Sucrose	+	+
Melibiose	+	+
Amygdalin	+	+

+, positive; −, negative; /, characteristic not found.

**Table 3:** Cellular fatty acid profiles of strain ZX-13 and CMYE1T.

Fatty Acid	ZX-13	CMYE1T
C12:0	3.47	4.2
C14:0	5.66	5.4
C16:0	37.46	32
C17:0	0.32	0.7
C17:0 cyclo	7.84	13.5
C19:0 cyclo ω8c	0.67	1.8
Summed feature 2	7.49	9
Summed feature 3	27.86	19.5
Summed feature 8	6.75	10.9

**Table 4:** ZX-13 genome assembly statistics and quality assessment.

Characteristics	Value
Sequence number	1
Total length	4,686,433
N50 length	4,686,433
Read coverage	1767.19
G + C content (%)	53.85
N rate (%)	0

**Table 5:** The general genome feature of strain ZX-13.

Feature	Value
Genome size (bp)	4,686,433
G + C content (%)	53.85
tRNA	86
5S rRNA	8
16S rRNA	7
23S rRNA	7
ncRNA	110
Total number of genes	4189
Genes assigned to NR	4030
Genes assigned to GO	2127
Genes assigned to KEGG	2719
Genes assigned to COG	3538
Genes assigned to Swiss-Prot	3212

**Table 6:** The potential gene clusters encoding secondary metabolites in ZX-13.

Type	Start	End	Similar Cluster	Similarity
NRPS	641,502	685,410	unknown	–
hserlactone	1,127,742	1,148,401	unknown	–
thiopeptide	1,489,613	1,515,804	O-antigen	14%
RiPP-like	1,907,458	1,917,655	unknown	–
terpene	2,335,363	2,358,975	carotenoid	100%
NRPS	2,765,675	2,843,718	hassallidin C	6%
NRP-metallophore	2,940,825	2,990,609	trichrysobactin/cyclic trichrysobactin/chrysobactin/dichrysobactin	53%
RRE-containing	3,882,783	3,903,067	lankacidin C	13%

–, similarity not found.

## Data Availability

The original contributions presented in this study are included in the article. Further inquiries can be directed to the corresponding author.

## References

[1] Prasannan P., Jeyaram Y., Pandian A., Raju R., Sekar S. (2020). A review on taxonomy, phytochemistry, pharmacology, threats and conservation of *Elaeocarpus* L. (Elaeocarpaceae). Bot. Rev..

[2] Bae G.Y., Lim M.W., Eom S.W., Lee H.L., Lee D.Y., Oh Y.J. (2023). Exploring the potential of Elaeocarpus sylvestris as natural biomaterials: In vitro antimicrobial and antioxidant properties, chemical constituents, and its effect on skin fibroblasts. Plant Biotechnol. Rep..

[3] Borase B.S., Surana S.S. (2024). A Comprehensive review on *Elaeocarpus floribundus* Blume. Curr. Tradit. Med..

[4] Habibah N.A., Nugrahaningsih N., Safitri S., Musafa F., Wijawati N. (2021). Profile of flavonoid and antioxidant activity in cell suspension culture of *Elaeocarpus grandiflorus*. Biosaintifika.

[5] Huang H.Y., Huang H.H., Zhao D.Y., Shan T.J., Hu L.L. (2022). *Pseudocryphonectria elaeocarpicola* gen. et sp. nov. (*Cryphonectriaceae, Diaporthales*) causing stem blight of *Elaeocarpus* spp. in China. MycoKeys.

[6] Guan L.L., Huang H.Y., Dai J., Gao C.L., Zhao Y., Wang Y.W. (2024). Biological characteristics of the pathogen for stem blight of *Elaeocarpus*. For. Environ. Sci..

[7] Chen T.T., Zhang Z.Z., Li W.Z., Chen J., Chen X.T., Wang B.C., Dai Y.Y., Ma J.L., Ding H.X., Wang W.Z. (2022). Biocontrol potential of *Bacillus subtilis* CTXW 7-6-2 against kiwifruit soft rot pathogens revealed by whole-genome sequencing and biochemical characterisation. Front. Microbiol..

[8] Xie L.Y., Liu L.F., Luo Y.J., Rao X.B., Di Y.N., Liu H., Qian Z.F., Shen Q.Q., He L.L., Li F.S. (2023). Complete genome sequence of biocontrol strain *Bacillus velezensis* YC89 and its biocontrol potential against sugarcane red rot. Front. Microbiol..

[9] Samaras A., Nikolaidis M., Antequera-Gómez M.L., Cámara-Almirón J., Romero D., Moschakis T., Amoutzias G.D., Karaoglanidis G.S. (2021). Whole genome sequencing and root colonization studies reveal novel insights in the biocontrol potential and growth promotion by *Bacillus subtilis* MBI 600 on cucumber. Front. Microbiol..

[10] Khalifa M.W., Rouag N., Bouhadida M. (2022). Evaluation of the antagonistic effect of *Pseudomonas rhizobacteria* on Fusarium wilt of chickpea. Agriculture.

[11] Ganeshan G., Kumar A.M. (2005). *Pseudomonas fluorescens*, a potential bacterial antagonist to control plant diseases. J. Plant Interact..

[12] Al-Ghafri H.M., Velazhahan R., Shahid M.S., Al-Sadi A.M. (2020). Antagonistic activity of *Pseudomonas aeruginosa* from compost against *Pythium aphanidermatum* and *Fusarium solani*. Biocontrol Sci. Technol..

[13] Soenens A., Imperial J. (2020). Biocontrol capabilities of the genus *Serratia*. Phytochem. Rev..

[14] Dandurishvili N., Toklikishvili N., Ovadis M., Eliashvili P., Giorgobiani N., Keshelava R., Tediashvili M., Vainstein A., Khmel I., Szegedi E. (2011). Broad-range antagonistic rhizobacteria *Pseudomonas fluorescens* and *Serratia plymuthica* suppress *Agrobacterium* crown gall tumours on tomato plants. J. Appl. Microbiol..

[15] Sharma A., Gupta A.K., Khosla K., Mahajan R., Bharti, Mahajan P.K. (2017). Antagonistic potential of native agrocin-producing non-pathogenic *Agrobacterium tumefaciens* strain UHFBA-218 to control crown gall in peach. Phytoprotection.

[16] Ciancio A. (2018). Biocontrol potential of *Pasteuria* spp. for the management of plant parasitic nematodes. CABI Rev..

[17] Kiani T., Mehboob F., Hyder M.Z., Zainy Z., Xu L., Huang L., Farrakh S. (2021). Control of stripe rust of wheat using indigenous endophytic bacteria at seedling and adult plant stage. Sci. Rep..

[18] Gardan L., Christen R., Achouak W., Prior P. (2004). *Erwinia papayae* sp. nov., a pathogen of papaya (*Carica papaya*). Int. J. Syst. Evol. Microbiol..

[19] López M.M., Rosello M., Llop P., Ferrer S., Christen R., Gardan L. (2011). *Erwinia piriflorinigrans* sp. nov., a novel pathogen that causes necrosis of pear blossoms. Int. J. Syst. Evol. Microbiol..

[20] Matsuura T., Mizuno A., Tsukamoto T., Shimizu Y., Saito N., Sato S., Kikuchi S., Uzuki S., Azegami K., Sawada H. (2012). *Erwinia uzenensis* sp. nov., a novel pathogen that affects European pear trees (*Pyrus communis* L.). Int. J. Syst. Evol. Microbiol..

[21] Rezzonico F., Smits T.H., Born Y., Blom J., Frey J.E., Goesmann A., Cleenwerck I., de Vos P., Bonaterra A., Duffy B. (2016). *Erwinia gerundensis* sp. nov., a cosmopolitan epiphyte originally isolated from pome fruit trees. Int. J. Syst. Evol. Microbiol..

[22] Du Y., Wang T., Lv C., Yan B., Wan X., Wang S., Kang C.Z., Guo L.P., Huang L. (2024). Whole Genome sequencing reveals novel insights about the biocontrol potential of *Burkholderia ambifaria* CF3 on *Atractylodes lancea*. Microorganisms.

[23] Teber R., Asakawa S. (2024). In silico screening of bacteriocin gene clusters within a set of marine *Bacillota* genomes. Int. J. Mol. Sci..

[24] Ferreira J.H.S., Matthee F.N., Thomas A.C. (1991). Biological control of *Eutypa lata* on grapevine by an antagonistic strain of *Bacillus subtilis*. Phytopathology.

[25] Pan M.K., Feng G.D., Yao Q., Li J.L., Liu C.J., Zhu H.H. (2022). *Erwinia phyllosphaerae* sp. nov., a novel bacterium isolated from phyllosphere of pomelo (*Citrus maxima*). Int. J. Syst. Evol. Microbiol..

[26] Dai P.B., Zong Z.F., Ma Q., Wang Y. (2019). Isolation, evaluation and identification of *rhizosphere actinomycetes* with potential application for biocontrol of *Valsa mali*. Eur. J. Plant Pathol..

[27] Emms D.M., Kelly S. (2015). OrthoFinder: Solving fundamental biases in whole genome comparisons dramatically improves orthogroup inference accuracy. Genome Biol..

[28] Chen C.J., Wu Y., Li J.W., Wang X., Zeng Z.H., Xu J., Liu Y.L., Feng J.T., Chen H., He Y.H. (2023). TBtools-II: A “one for all, all for one” bioinformatics platform for biological big-data mining. Mol. Plant.

[29] Vanneste J.L., Yu J. (1995). Biological control of fire blight using *Erwinia herbicola* Eh252 and *Pseudomonas fluorescens* A506 separately or in combination. VII International Workshop on Fire Blight 411.

[30] Kempf H.J., Wolf G. (1989). *Erwinia herbicola* as a biocontrol agent of *Fusarium culmorum* and *Puccinia recondita* f. sp. *tritici* on wheat. Phytopathology.

[31] Bi W.L., Wang R., Yang Y.Y., Wang Y., Ma Z.T., Wang Q., Zhang D.F. (2021). *Pantoea vagans* strain BWL1 controls blue mold in mandarin fruit by inhibiting ergosterol biosynthesis in *Penicillium expansum*. Biol. Control.

[32] Kim W.I., Choi S.Y., Han I., Cho S.K., Lee Y., Kim S., Kang B., Choi O., Kim J. (2020). Inhibition of *Salmonella enterica* growth by competitive exclusion during early alfalfa sprout development using a seed-dwelling *Erwinia persicina* strain EUS78. Int. J. Food Microbiol..

[33] Maslova T.G., Markovskaya E.F., Slemnev N.N. (2021). Functions of carotenoids in leaves of higher plants. Biol. Bull. Rev..

[34] Smits T.H., Rezzonico F., Kamber T., Goesmann A., Ishimaru C.A., Stockwell V.O., Frey E.J., Duffy B. (2010). Genome sequence of the biocontrol agent *Pantoea vagans* strain C9-1. J. Bacteriol..

[35] Knirel Y.A., Bystrova O.V., Kocharova N.A., Zähringer U., Pier G.B. (2006). Conserved and variable structural features in the lipopolysaccharide of *Pseudomonas aeruginosa*. J. Endoxtin Res..

[36] Humisto A., Jokela J., Teigen K., Wahlsten M., Permi P., Sivonen K., Herfindal L. (2019). Characterization of the interaction of the antifungal and cytotoxic cyclic glycolipopeptide hassallidin with sterol-containing lipid membranes. Biochim. Biophys. Acta-Biomembr..

[37] Cai L., Seiple I.B., Li Q. (2021). Modular chemical synthesis of streptogramin and lankacidin antibiotics. Acc. Chem. Res..

